# The linkage between Bitcoin and foreign exchanges in developed and emerging markets

**DOI:** 10.1186/s40854-023-00454-w

**Published:** 2023-01-16

**Authors:** Ahmed BenSaïda

**Affiliations:** 1 LaREMFiQ Laboratory, University of Sousse, Sousse, Tunisia; 2 College of Business, Effat University, Jeddah, Saudi Arabia

**Keywords:** Cryptocurrency, Fiat currency, Bitcoin crashes, Market integration, C58, G11, G15, F31

## Abstract

This study investigates the connectedness between Bitcoin and fiat currencies in two groups of countries: the developed G7 and the emerging BRICS. The methodology adopts the regular (R)-vine copula and compares it with two benchmark models: the multivariate *t* copula and the dynamic conditional correlation (DCC) GARCH model. Moreover, this study examines whether the Bitcoin meltdown of 2013, selloff of 2018, COVID-19 pandemic, 2021 crash, and the Russia-Ukraine conflict impact the linkage with conventional currencies. The results indicate that for both currency baskets, R-vine beats the benchmark models. Hence, the dependence is better modeled by providing sufficient information on the shock transmission path. Furthermore, the cross-market linkage slightly increases during the Bitcoin crashes, and reaches significant levels during the 2021 and 2022 crises, which may indicate the end of market isolation of the virtual currency.

## Introduction

Over the last few years, investors and scholars have paid particular attention to cryptocurrencies. Some have focused on the emerging technology, while others are increasingly focusing on their ability to generate high yields. Irrespective of the focus, cryptocurrencies should be regarded as a fascinating topic for economic and financial researchers due to their significant capacity to undermine financial stability, payment systems, and even monetary ones (Böhme et al. 2015).

Cryptocurrencies quickly attracted the attention of investors who are seeking new international monetary alternatives, and of traders and hedgers who are looking for better investment opportunities. The success of Bitcoin has intensified financial institutions’ awareness of the importance of decentralized currencies, and has led to the emergence of a plethora of other cryptocurrencies known as altcoins.

Nevertheless, a synopsis of the literature highlights an incomplete picture of the dependency structure between the Bitcoin market and foreign exchange (forex) markets. Although numerous researchers have extensively examined financial market linkages over the last few decades, the study of the dynamics between cryptocurrencies and fiduciary currencies is still embryonic.

A good understanding of co-movements of currency prices across international markets is of great importance to market participants. Indeed, their investment strategies could be more profitable if market correlations are well understood. In this context and to address this gap, this study examines the dependence structure between Bitcoin and two sets of exchange rates: the developed G7 and the emerging BRICS markets.

Several studies have investigated the developed G7 and emerging BRICS markets, including Mensi et al. (2021), who compared the volatility spillovers between strategic commodity futures markets (oil and gold) and G7 and BRICS stock markets. Moreover, Shahzad et al. (2022b) argue that BRICS countries have acquired important roles in the world economy through international trade and financial and economic output. In this sense, Zhang and Hamori (2022) examined the connectedness between BRICS geopolitical risk and the U.S. macro economy and found evidence that shocks from the former have an impact on emerging economies. Regarding the BRICS forex markets, Salisu et al. (2022) examined the predictability of geopolitical risk for exchange rate volatility, and Xu and Lien (2022) analyzed forex market dependence during COVID-19. Concerning the effect of cryptocurrencies on BRICS, Dahir et al. (2020) examined the volatility dynamic connectedness between Bitcoin and emerging stock markets and concluded that Bitcoin volatility transmission is not an important source of shocks in BRICS equity markets. Goodell et al. (2022) investigated the impact of BRICS regulatory announcements on cryptocurrency volatilities and returns, and discovered important linkages between emerging markets and cryptocurrencies. Consequently, in the recent financial literature, BRICS countries have garnered considerable interest alongside developed G7 countries.

This study seeks to present a crucial interest for risk-seeking investors who need to know if and in what way cryptocurrencies and fiat currencies are linked, so they can assess the risk of their investment portfolios more accurately, and reap the advantages of diversification. More precisely, this study addresses the following questions: (a) do cryptocurrencies affect the fiat currencies in the same way for developed and emerging forex markets? (b) What are the dependence dynamics among digital and traditional currencies? Is it better described as a whole structure, or is the information on the shock transmission path is as important as the dependence itself? (c) How can investors from developed and emerging countries benefit from the cross-linkage between virtual and conventional markets when constructing international portfolios? The answers contribute to the financial literature on cryptocurrencies as they reveal the ambiguous connectedness between both markets.

This study adopts the general theoretical framework of regular vine copula and deals with several well-documented Bitcoin crashes, namely, the 2013 meltdown, 2018 selloff, COVID-19 pandemic, 2021 crash, and the Russia-Ukraine conflict in 2022, to investigate the effect of cryptocurrency on fiat currencies. Based on a sample from April 28, 2013 to September 10, 2022, the main results suggest that the Bitcoin market remains isolated from conventional foreign exchange markets, even during crash events, and exhibits a safe haven property in both developed and emerging forex markets. However, during the 2021 and 2022 crashes, the cross-market linkage between Bitcoin and fiat currencies significantly increased, which indicates the beginning of market integration of the digital currency.

This study contributes to the existing literature in two ways. (1) First, it examines the dependency structure between Bitcoin and the main exchange rates using copulas. This investigation reveals the type of connectedness between virtual and conventional markets, as well as the shock transmission path across currencies. (2) Second, this study investigates the impact of Bitcoin crashes on fiduciary currencies. The analysis of the turmoil of digital currency reveals the degree of market integration. In summary, this study concerns both local and global investors who are active in cryptocurrency and forex exchange markets. Moreover, monetary authorities could benefit from this study by implementing appropriate procedures to prevent harmful shocks to forex markets.

The remainder of this paper is organized as follows. "Literature review" Section provides a broad conceptual overview of the literature on the Bitcoin market. "Theoretical framework" Section surveys the theoretical framework and methodology. "Empirical investigation" Section summarizes the data and conducts the empirical investigation. "Conclusion" Section presents concluding remarks.

## Literature review

A cryptocurrency is a peer-to-peer electronic cash system that allows online transactions without resorting to a financial intermediary (see Xu et al. 2019, forareviewonblockchaintechnology). Unlike conventional currencies, it is a fully decentralized system over which neither governments nor central authorities have control. Since the launch of Bitcoin by Nakamoto (2008), cryptocurrencies have gained considerable traction in exchange markets as economic instruments and have become increasingly relevant. Bitcoin has become widespread due to the failure of central banks in the wake of the global financial crisis of 2007–2008. In fact, the Bitcoin universe continues to be one of the most attractive growth stories in recent years. Since the creation of this digital currency in 2008, the number of Bitcoins in circulation has been steadily growing, reaching approximately 17.3 million in September 2018 according to coinmarketcap. Currently, the market capitalization of the world’s top virtual currency is more than $112 billion, representing approximately 50% of the total cryptocurrency market capitalization. At the end of 2013, its price was only about $350, whereas in 2018, one Bitcoin reached approximately $7305 (see, Fang et al. 2022, forareviewoncryptocurrencytrading). However, Bitcoin has faced several crashes over the last few years, notably the 2013 Bitcoin price crash, 2018 selloff, COVID-19 pandemic, 2021 crash, and the latest Russia-Ukraine conflict.

In this vein, some researchers are interested in studying the Bitcoin concept to understand the structure and functioning of this system, which could be a means of prevention in the future and help anticipate shocks and reduce damage caused by price crashes (Böhme et al. 2015).

There have been several discussions about whether Bitcoin meets the standard properties of a real currency or is just a speculative asset. For instance, Bouoiyour et al. (2015) demonstrate that Bitcoin has no fundamental value and does not act much in the same way as a currency but rather as speculative foolery. However, Kristoufek (2015) argues that, in addition to being a purely speculative asset, Bitcoin represents a standard financial asset. Hanley (2018) reveals that Bitcoin does not have an intrinsic value; hence, it cannot compete against conventional currencies. Nevertheless, Woo et al. (2013) indicate that Bitcoin exhibits several money-like properties, making it possible to assign fair value to this digital currency. Garcia et al. (2014) state that production costs could add some intrinsic value to Bitcoins. Furthermore, Baur and Dimpfl (2021) ascertain that Bitcoin cannot work as a currency because it is unstable and not backed by any government. Nevertheless, it has the characteristic of a store of value over long horizons.

Other scholars have pointed to the financial characteristics of Bitcoin. Popper (2016) regards Bitcoin as digital gold and Bouri et al. (2017a) underline some properties as an investment vehicle, especially the capability to be a safe haven. Bouri et al. (2017b) reveal that Bitcoin represents an effective diversifier but a poor hedge. Dyhrberg (2016) highlights the hedging capabilities of Bitcoin. In this spirit, Bouri et al. (2017a) also confirm that Bitcoin exhibits strong hedge and safe haven abilities in comparison with general commodity indices and energy indices. Moreover, Baur and Lucey (2015) investigate the statistical properties of Bitcoin and establish that it exhibits low correlation with traditional asset classes such as commodities, stocks, bonds, and currencies in normal periods as well as during times of financial crises.

Additionally, several researchers focus on Bitcoin price discovery. For example, Brandvold et al. (2015) investigate how the different exchange platforms can contribute to the price formation of Bitcoin, and demonstrate that BTC-e and Mt. Gox are the major price leaders in the market as they have the highest information share of exchange. In this context, Bouoiyour et al. (2015) and Kristoufek (2015) indicate that there is strong bidirectional causality between Bitcoin prices and search requests for the word “Bitcoin” using tools such as Google Trends and the frequency of visits of this term on Wikipedia. Polasik et al. (2015) note that Bitcoin returns increase as the volume of queries for this word on Google increases. Moreover, they found that popularity, the volume of transactions, and the number of newspaper articles on this cryptocurrency are in some way driving its price. Kristoufek (2015) reveals that the price of Bitcoin is considerably driven by the Trade Exchange ratio, and Ciaian et al. (2016) demonstrate that supply/demand fundamentals and Bitcoin’s attractiveness significantly affect its price formation, especially the demand side drivers, the total number of Bitcoin transactions, and the velocity of circulation. Garcia et al. (2014) investigate the role of social interactions in the formation of Bitcoin price bubbles. Sebastião and Godinho (2021) study the predictability and the profitability of trading strategies of major cryptocurrencies using machine learning.

Several studies focus on the diversification opportunities provided by this virtual currency. For example, Brière et al. (2015) find that Bitcoin needs to be included in well-diversified investment portfolios since it can improve their risk/return profile. In fact, there is a very low correlation between Bitcoin and other traditional assets; hence, it is considered a good diversifier. In this sense, Kurka (2019) argues that the connectedness between Bitcoin and other assets, namely S &P 500, oil, gold, Japanese yen, and Euro, is very weak. Corbet et al. (2018) and Ji et al. (2018) underscore that cryptocurrencies are characterized by being isolated from economic and financial assets.

In the wake of the growing interest in cryptocurrencies, researchers have vigorously debated further questions about Bitcoin. For instance, Cheah and Fry (2015) shed light on the presence of speculative bubbles in the Bitcoin market as with other financial markets. They also determine that Bitcoin presents an intrinsic value equal to zero. Blau (2017) finds that speculative trading is not responsible for the unusually high level of Bitcoin’s volatility, suggesting that it is considered a currency rather than a speculative asset.

Other studies discuss Bitcoin within the framework of existing alternative monetary systems. For instance, Shubik (2014) proves that, compared to the alternative electronic systems, such as debit and credit cards, Bitcoin helps stimulate the global economy while being in line with government concerns regarding taxation and illegal activities. Rogojanu and Badea (2014) compare Bitcoin to alternative private currency and to traditional currencies. In this regard, Carrick (2016) does not consider Bitcoin as a substitute for fiat currencies but rather as a complement to emerging market currencies. Furthermore, Levulytè and Šapkauskiené (2021) investigate the ability of cryptocurrencies to fulfill three main monetary functions: medium of exchange, unit of account, and store of value.

Researches on the link between digital currencies and forex markets include both theoretical and empirical examinations. For instance, Corbet et al. (2017) argued that Bitcoin is subject to the same economic factors as traditional fiat currencies, and is not entirely unaffected by government policies. Kang and Lee (2022) constructed a search-theoretic model to investigate how conventional money and Bitcoin facilitate transactions and concluded that benefits in an economy with both is lower than in a money-only economy due to surcharges in the confirmation of Bitcoin transactions. From an empirical viewpoint, Aharon et al. (2021) examined the connectedness between the volatility of Bitcoin and the exchange rates of the main fiat currencies and revealed that Bitcoin is independent from any main currency; hence, it may provide hedging benefits. Other recent studies investigated the linkage between cryptocurrencies and fiat currencies, including Rognone et al. (2020), Chemkha et al. (2021a), Huang et al. (2022), Shahzad et al. (2022a), Virk (2022), among others.

## Theoretical framework

In the financial literature, two major approaches have been identified to investigate the dependence between financial markets: (1) the multivariate GARCH (MGARCH) models based on the dynamic conditional linear correlation (DCC) (e.g., Urquhart and Zhang 2019; Virk 2022) or BEKK ( Palazzi et al. 2021, amongothers), and (2) the copula theory, which, as Ning (2010) highlights, is capable of detecting nonlinear and asymmetric dependency. Other approaches based on traditional correlations have been highly criticized due to their low performance to detect complex dependence dynamics among variables. The copula theory has the advantage of detecting the shock transmission path among variables, a desired property that multivariate GARCH models lack, as discussed by BenSaïda and Litimi (2021). Therefore, the current study prefers to model the multivariate dependence using the copula framework.

### Copula theory background

Over the past few decades, there has been growing interest in modeling dependence using copulas. Indeed, scholars have identified many successful applications in several fields. More accurately, the copula function has been used for several purposes due to its flexibility. This section introduces some useful definitions and properties related to copulas.

A copula function expresses the joint distribution of two or more random variables. With copulas, the joint distribution can be separated into two quantities: the marginal distribution of each variable and the copula that combines these marginals into a joint distribution.

Sklar (1959) theorem states that for any *d*-dimensional random vector ${\varvec{x}}=\left( x_1, \ldots , x_d \right) \in {\mathbb{R}}^d$ with joint cumulative distribution function (*cdf*) $F\left( x_1, x_2, \ldots , x_d\right)$ and continuous marginal distribution functions $F_i(x_i)$, for $i \in \left\{ 1, 2, \ldots , d\right\}$; then, a unique *d*-dimensional copula function $C \in \left[ 0,1 \right] ^d$ exists such that the multivariate distribution function is written in terms of univariate marginals $F_i$, and each marginal $F_i \left( x_i \right) = u_i$ is independent and uniformly distributed on $\left[ 0,1 \right]$:

1 $$\begin{aligned} F \left( x_1,\ldots ,x_d \right) = C \left( \underbrace{F_1 \left( x_1 \right) }_{u_1}, \ldots , \underbrace{F_d \left( x_d \right) }_{u_d} \right) \end{aligned}$$

From Eq. (1), the copula can be constructed according to the following formula:

2 $$\begin{aligned} C \left( u_1,\ldots , u_d \right) = F \left( F^{-1}_1 \left( u_1 \right) , \ldots , F^{-1}_d \left( u_d \right) \right) \end{aligned}$$

where $F^{-1}_i$ are the inverse distribution functions of marginals.

The contribution of Sklar (1959) theorem is that it separates the modeling of the marginal distributions $F_i \left( x_i \right)$ from the copula. If the above joint cumulative distribution function *F* is *d*-times differentiable, then the joint probability distribution function (*pdf*) can be derived as follows:

3 $$\begin{aligned} \underbrace{f \left( x_1, x_2, \ldots , x_d \right) }_{f \left( {\varvec{x}} \right) } = {}&\frac{\partial ^d }{\partial x_1 \ldots \partial x_d} F \left( x_1, \ldots , x_d \right) \nonumber \\ = {}&\prod \limits _{i=1}^d f_i\left( x_i \right) \frac{\partial ^d }{\partial u_1 \ldots \partial u_d} C \left( u_1, \ldots , u_d \right) \nonumber \\ = {}&\prod \limits _{i=1}^d f_i\left( x_i \right) c \left( u_1, \ldots , u_d \right) \end{aligned}$$

The *pdf* of the copula is:

4 $$\begin{aligned} c \left( u_1,\ldots , u_d \right) = \frac{f \left( F^{-1}_1 \left( u_1 \right) , \ldots , F^{-1}_d \left( u_d \right) \right) }{\prod \limits _{i=1}^d f_i\left( F^{-1}_i \left( u_i \right) \right) } \end{aligned}$$

An appealing feature of copula functions is that they offer a complete separation between the marginals and the dependence structure (BenSaïda and Litimi 2021). The estimation of the marginals is quite straightforward and requires standard univariate techniques. Nevertheless, the estimation of the dependence structure requires more advanced methods. For instance, for the bivariate case, a copula can be selected from a large collection of two-dimensional functions, such as Gaussian, Clayton, Frank, or Gumbel. However, for the multivariate case, the choice among copula families becomes limited. The literature proposes the pair copula construction (PCC) technique based on bivariate functions as building blocks, or vine copula.

### Modeling the marginals

Several time-series models can explain a few stylized facts common to most financial data, such as volatility clustering, leptokurtosis, and the leverage effect. Hence, to produce a series of independent and identically distributed (*i.i.d.*) observations, the GJR model of Glosten et al. (1993) is fitted to the returns of each variable $r_{i,t}$, where $i \in \left\{ 1, \ldots , d\right\}$ and $t \in \left\{ 1, \ldots , T\right\}$ for the sample size *T*. The GJR model considers the asymmetric leverage effect and leptokurtosis in the conditional variance behavior. Thus, the GJR of orders (*p*, *q*) process is given by:

5 $$\begin{aligned} r_{i,t} = {}&\mu _{i,t} + \epsilon _{i,t} \nonumber \\ \epsilon _{i,t} = {}&z_{i,t} \sqrt{h_{i,t}} \text {, where }z_{i,t}\text { is a white noise process.} \nonumber \\ h_{i,t} = {}&\kappa _i + \sum \limits _{l=1}^q \left( \gamma _{i,l} + \alpha _{i,l} {{1}}_{\left[ \epsilon _{i,t-l} < 0 \right] } \right) \epsilon _{i,t-l}^2 + \sum \limits _{s=1}^p \beta _{i,s} \, h_{i,t-s} \end{aligned}$$

where $\mu _{i,t}$ represents the conditional mean that may include a constant and/or past observations. The coefficients $\kappa _i >0$, $\gamma _{i,l} \geqslant 0$, $\alpha _{i,l} \geqslant 0$, $\beta _{i,s} \geqslant 0$, and $z_{i,t}$ are a sequence of *i.i.d.* random variables with zero mean and unit variance; $h_{i,t}$ represents the conditional variance of the process.

The input $u_{i,t}$ to the copula model for each variable *i* and observation *t* are the uniformly distributed probability integral transforms derived from the marginals as $u_{i,t} = F_i \left( \frac{\epsilon _{i,t}}{\sqrt{h_{i,t}}} \right)$, where $F_i$ represents the cumulative distribution function of the white noise process $z_{i,t}$ (BenSaïda 2018).

### Regular vines

In the methodology, the regular (R) vine decomposition is employed since it offers a great deal of flexibility in the dependence structure (see, BenMim and BenSaïda 2019, fordetaileddiscussion). R-vines have no prior specific form, the structure of the trees is derived according to the existing relationship among variables.

A total of two special cases of the regular vine copula exist, the canonical (C) vine and the drawable (D) vine. Both decompositions have a pre-determined dependence structure. For instance, a C-vine has a star structure, where a central dominant variable governs the interactions between all other variables. However, a D-vine has a path structure that resembles a one-way direction linking all variables in a row.

The choice of R-vine decomposition is justified by the fact that it offers a powerful environment to study the dependence structure. Moreover, the vine copula has many desirable properties compared to other copula constructions, such as flexible and wide-range dependence, flexible tail asymmetry, and flexible tail dependence, among others (BenSaïda and Litimi 2021).

### Benchmark models

Several models have studied the dependence dynamics among variables, mainly multivariate GARCH and copula-based models. Consequently, the results of the selected R-vine decomposition are compared to those of two benchmark models. (1) First, the multivariate Student’s $t_\nu$ copula, where the dependence is modeled as a whole, disregarding the shock transmission path. (2) Second, the multivariate DCC-GARCH model, which detects the linear dependency among variables ruling out any nonlinear connectedness.

#### Multivariate student’s $t_\nu$ copula

The symmetric *d*-variate $t_\nu$ density with correlation matrix ${\mathbf{R}}$ and for ${\varvec{x}} \in {\mathbb{R}}^d$ is defined in Eq. (6), where $\nu$ denotes the degrees-of-freedom.

6 $$\begin{aligned} t_{d,\nu } \left( {\varvec{x}}, {\mathbf{R}} \right) = \left| {\mathbf{R}} \right| ^{-\frac{1}{2}} \frac{\Gamma \left( \frac{\nu +d }{2}\right) }{\Gamma \left( \frac{ \nu }{2}\right) \left( \pi \, \nu \right) ^\frac{d}{2}} \left( 1+ \frac{{\varvec{x}}^{\prime} {\mathbf{R}}^{-1} {\varvec{x}}}{\nu } \right) ^{-\frac{\nu +d}{2}} \end{aligned}$$

Therefore, the multivariate $t_\nu$ copula cumulative distribution function for ${\varvec{u}} \in \left[ 0,1\right] ^d$ is:

$$\begin{aligned} C\left( {\varvec{u}}, {\mathbf{R}}, \nu \right) = T_{d,\nu } \left( T^{-1}_{1,\nu } \left( u_1 \right) , \ldots , T^{-1}_{1,\nu } \left( u_d \right) ; {\mathbf{R}} \right) \end{aligned}$$

where $T_{d,\nu } \left( \cdot \right)$ is the multivariate *cdf* of the Student’s $t_\nu$, and $T^{-1}_{1,\nu } \left( \cdot \right)$ is the univariate inverse *cdf*. The multivariate $t_\nu$ copula density is expressed as follows,^1^

$$\begin{aligned} c\left( {\varvec{u}}, {\mathbf{R}}, \nu \right) = \frac{t_{d,\nu } \left( T^{-1}_{1,\nu } \left( u_1 \right) , \ldots , T^{-1}_{1,\nu } \left( u_d \right) ; {\mathbf{R}} \right) }{\prod _{j=1}^{d} t_{1,\nu }\left[ T^{-1}_{1,\nu } \left( u_j \right) \right] } \end{aligned}$$

#### Multivariate DCC model

The *d*-dimensional returns are modeled as:

$$\begin{aligned} \begin{aligned} {\varvec{r}}_t = {}&{\varvec{\mu}}_t + {\varvec{\epsilon}}_t \\ {\varvec{\epsilon}}_t = {}&{\mathbf{H}}_{t}^{{1}/{2}} {\varvec{z}}_t \end{aligned} \end{aligned}$$

where ${\varvec{\mu}}_t$ represents the conditional mean vector that may include constants and/or past observations. The quantity ${\varvec{\epsilon}}_t$ denotes a $(d \times 1)$ vector of residuals and ${\varvec{z}}_t$ is a $(d \times 1)$ *i.i.d.* random vector of errors. ${\mathbf{H}}_t$ represents a $(d \times d)$ conditional covariance matrix of ${\varvec{r}}_t$ such that:

$$\begin{aligned} {\mathbf{H}}_t = {\mathbf{D}}_t \, {\mathbf{R}}_t \, {\mathbf{D}}_t \end{aligned}$$

where ${\mathbf{R}}_t$ represents the time-varying conditional correlation matrix and ${\mathbf{D}}_t$ denotes a $(d \times d)$ diagonal matrix containing the conditional standard deviations of univariate GARCH-type models, such that:

$$\begin{aligned} \begin{aligned} {\mathbf{D}}_t = {}&\mathrm {diag} \left( h_{1,t}^{{1}/{2}}, \ldots , h_{d,t}^{{1}/{2}}\right) \\ {\mathbf{R}}_t = {}&\mathrm {diag} \left( {\mathbf{Q}}_t \right) ^{-{1}/{2}} \, {\mathbf{Q}}_t \, \mathrm {diag} \left( {\mathbf{Q}}_t \right) ^{-{1}/{2}} \end{aligned} \end{aligned}$$

The term ${\mathbf{Q}}_t$ is a positive-definite matrix that represents the evolution of the conditional correlation of the standardized residuals ${\varvec{z}}_t$ such that ${\mathbf{Q}}_t = \left\{ q_{ij,t} \right\} _{i,j = 1}^d$. A typical element of ${\mathbf{R}}_t$ has the form $\rho _{ij,t} = \frac{q_{ij,t}}{\sqrt{q_{ii,t} \; q_{jj,t}}}$, where $\rho _{ij,t}$ denotes the correlation estimator. The dynamics of ${\mathbf{Q}}_t$ for the DCC(*p*, *q*) model are,

$$\begin{aligned} {\mathbf{Q}}_t = {\bar{\mathbf{Q}}} + \sum \limits _{l=1}^q a_l \left( {\varvec{z}}_{t-l} {\varvec{z}}_{t-l}' - {\bar{\mathbf{Q}}} \right) + \sum \limits _{s=1}^p b_s \left( {\mathbf{Q}}_{t-s} - {\bar{\mathbf{Q}}} \right) \end{aligned}$$

The DCC(1,1) model can be expressed in Eq. (7).

7 $$\begin{aligned} {\mathbf{Q}}_t = \left( 1 - a - b \right) {\bar{\mathbf{Q}}} + a {\varvec{z}}_{t-1} {\varvec{z}}_{t-1}' + b {\mathbf{Q}}_{t-1} \end{aligned}$$

The coefficients *a* and *b* are non-negative scalars that capture the effects of previous shocks and previous conditional correlations, respectively, on the current conditional correlation. A necessary and sufficient condition for ${\mathbf{Q}}_t$ to be positive-definite is that $a + b < 1$. The quantity ${\bar{\mathbf{Q}}}$ is the unconditional covariance matrix of the standardized residuals $z_{i,t} = \frac{\epsilon _{i,t}}{\sqrt{h_{i,t}}}$ such that ${\bar{\mathbf{Q}}} = {\mathbb{E}} \left( {\varvec{z}}_t {\varvec{z}}_t^\prime \right)$. In practice, the expectation of ${\bar{\mathbf{Q}}}$ is infeasible; hence, it is replaced with the sample analog $\frac{1}{T} \sum \limits _{t=1}^T {\varvec{z}}_t {\varvec{z}}_t'$.

The DCC model is estimated using a two-step maximum likelihood method (Chemkha et al. 2021b). (1) In the first step, the conditional variances are estimated separately using a univariate GARCH-type model for each time series using Eq. (5). (2) In the second step, the conditional correlation in Eq. (7) is estimated by assuming that the standardized residuals ${\varvec{z}}_t$ follow a multivariate Student’s *t* distribution $z_{t} \mathop \sim \limits ^{{i.id.}} t_{{d,v}}$(**0**,** R**_t_) (see, Urquhart and Zhang 2019; Chemkha et al. 2021b, fordetaileddevelopment).^2^

## Empirical investigation

This section determines the most appropriate copula that captures the dependence structure between Bitcoin and the G7 exchange rates and between Bitcoin and the BRICS exchange rates.

First, the returns are filtered using a GJR model to obtain the standardized uniform residuals of each currency rate returns. Then, given the filtered residuals, the R-vine structure is estimated and compared to the benchmark models, i.e., the multivariate $t_\nu$ copula and the DCC-GARCH model. Finally, the best copula that fits the data is selected using the Bayesian information criterion (BIC) and Akaike information criterion (AIC), where the preferred model has the lowest criteria.^3^

### Data and summary statistics

The dataset consists of daily exchange rates of Bitcoin (BTC) and nine government currencies, which are the developed G7 countries (U.S., Canada, France, Germany, Italy, U.K., and Japan), and the emerging BRICS (Brazil, Russia, India, China, and South Africa). All currencies are quoted against the U.S. dollar (USD), since the USD is the most traded currency around the world and is often considered the comparison standard for currencies. Therefore, one exchange rate corresponds to one unit of a foreign currency in terms of the USD. This is a typical choice in the forex literature, where any potential shock in the U.S. market is integrated into pairwise exchange rates.

The sample period spans from April 28, 2013 to September 10, 2022, yielding a total of 3420 daily observations. Bitcoin data were downloaded from coinmarketcap and fiat currency data from Dukascopy Swiss Forex Bank online database. The collected data correspond to the exchange rates at exactly the same time, 00:00 GMT, to remove any potential problems of asynchronous data, where the time measurement differs in markets with different trading hours.

**Fig. 1:**
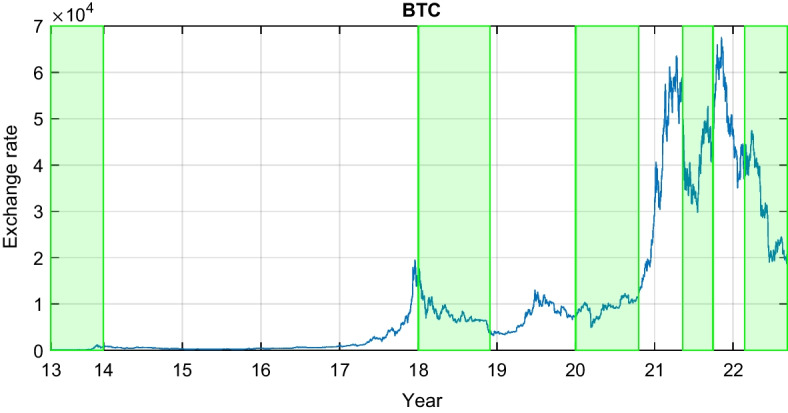
Bitcoin exchange rate quoted against the USD. The shaded areas correspond to the main Bitcoin crashes documented in the financial literature, mainly, the 2013 meltdown, the 2018 selloff, the COVID-19 pandemic, the 2021 crash, and the Russia-Ukraine conflict in 2022

**Fig. 2:**
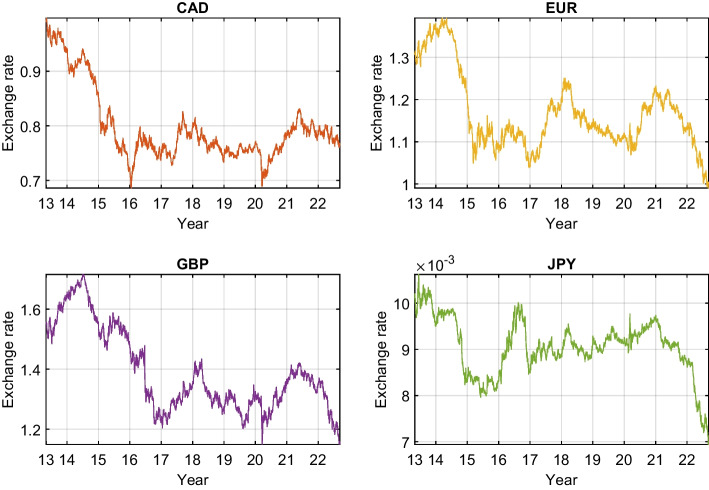
The G7 exchange rates quoted against the USD

**Fig. 3:**
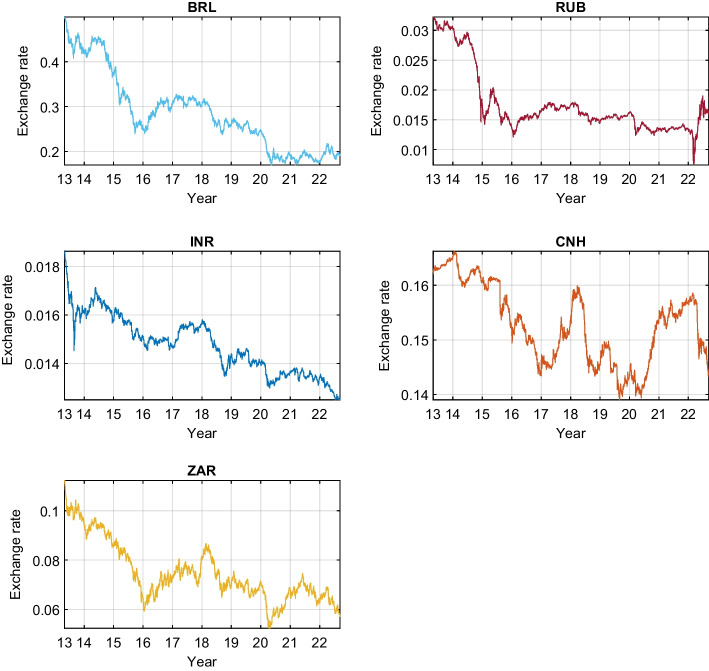
The BRICS exchange rates quoted against the USD

Regarding the Chinese currency, this study selects the offshore Renminbi (or Yuan) CNH, which is freely traded on the global markets outside mainland China. The exchange rate of CNH is decided by the free market and does not face the same governmental restrictions as the onshore Yuan CNY. Table 1 displays the tickers of the two groups of currencies. Figure 1 plots the evolution of Bitcoin during the sample period with shaded main turmoil episodes. Figures 2 and 3 exhibit the G7 and BRICS exchange rates, respectively.

**Table 1:** Currency tickers

G7 currencies		BRICS currencies	
USD	U.S. dollar	BRL	Brazilian real
EUR	Euro	RUB	Russian ruble
CAD	Canadian dollar	INR	Indian rupee
GBP	British pound	CNH	Offshore Chinese yuan
JPY	Japanese yen	ZAR	Zuid-Afrikaans rand

This table presents the abbreviations of the studied exchange rates

To deal with potential non-stationarity of currency rates usually detected in financial data, all variables are transformed into returns by taking the logarithmic difference of the daily exchange rates: $r_t = \ln S_t - \ln S_{t-1}$. Table 2 reports the summary statistics for the daily returns.

**Table 2:** Descriptive statistics of BTC, G7 and BRICS exchange rate returns

	Panel A				
	BTC	CAD	EUR	GBP	JPY
Mean	0.0015$^\dag$	− 7.4E−5	− 7.7E−5	− 8.6E−5	−0.0001
Std. dev.	0.0418	0.0039	0.0041	0.0047	0.0046
Minimum	$-$0.4647	$-$0.0209	$-$0.0257	$-$0.0638	$-$0.0314
Maximum	0.3575	0.0197	0.0302	0.0287	0.0395
Skewness	$-$0.5006	$-$0.0541	0.0188	$-$0.8988	0.2669
Kurtosis	13.742	5.9798	7.5645	17.788	11.420
J-B stat.	16528*	1261.0*	2957.0*	31513*	10107*

	Panel B				
	BRL	RUB	INR	CNH	ZAR
Mean	$-$0.0003	$-$0.0002	$-$0.0001$^\dag$	$-$3.5E-5	$-$0.0002
Std. dev.	0.0088	0.0164	0.0034	0.0022	0.0083
Minimum	$-$0.0712	$-$0.4610	$-$0.0373	$-$0.0273	$-$0.0495
Maximum	0.0592	0.4276	0.0336	0.0147	0.0508
Skewness	$-$0.0843	$-$1.8763	$-$0.6888	$-$0.3729	$-$0.2801
Kurtosis	7.1953	335.07	17.990	15.842	5.7631
J-B stat.	2502.0*	1.56E7*	32181*	23498*	1128.0*

This table reports the descriptive statistics of the exchange rate returns

$\dag$ Mean is statistically not different from zero at the 5% significance level

* Normality is rejected at the 5% significance level

According to Table 2, the means of the returns are negative, but very close to zero, except for BTC. Bitcoin return presents the highest standard deviation, while CNH has the lowest, which implies that Bitcoin is highly volatile. Indeed, Baur and Dimpfl (2021) demonstrate that the volatility of Bitcoin is extreme and higher than that of the major exchange rates. Moreover, all currencies (except EUR and JPY) exhibit negative skewness, which means that the tail of the return distribution is longer on the left-hand side. The kurtosis coefficients are above 3 for all variables. The Jarque-Bera test rejects the normality of all returns at the 5% significance level.

Table 3 presents the pairwise linear correlations between Bitcoin and the fiat currency returns. The correlation coefficients between Bitcoin and other currency returns are positive and statistically significant for all G7 and BRICS currency returns (except for BRL, INR, and ZAR). Nevertheless, the pairwise relationships are negligible in terms of magnitude because the coefficients are less than 0.05 (except for CNH, where the coefficient is around 0.07). Consequently, there is insufficient evidence that the digital market is integrated with the forex market, and it might be isolated during the entire study period.

**Table 3:** Correlation matrices

	Panel A: G7 basket				
	BTC	CAD	EUR	GBP	JPY
BTC	1.0000				
CAD	0.0416**	1.0000			
EUR	0.0486***	0.3933***	1.0000		
GBP	0.0328*	0.1073***	0.1265***	1.0000	
JPY	0.0337**	0.1194***	0.4185***	0.0547***	1.0000

	Panel B: BRICS basket					
	BTC	BRL	RUB	INR	CNH	ZAR
BTC	1.0000					
BRL	0.0197	1.0000				
RUB	0.0305*	0.0150	1.0000			
INR	0.0255	0.2156***	0.0522***	1.0000		
CNH	0.0715***	0.0497***	0.1262***	0.0203	1.0000	
ZAR	0.0141	− 0.0148	0.2049***	− 0.0304*	0.2842***	1.0000

This table reports the Pearson’s linear correlation matrices between Bitcoin and G7 basket returns (Panel A), and between Bitcoin and BRICS basket returns (panel B) from April 28, 2013 to September 10, 2022

*** Significantly different from zero at the 1% significance level

** Significantly different from zero at the 5% significance level

* Significantly different from zero at the 10% significance level

### Marginal estimation

Several GARCH-type models can be used to model marginal distributions and fit historical return data. In this case, this study selects a GJR-GARCH (1,1) model under the Student’s *t* distribution because it considers the stylized facts exhibited by financial returns, such as volatility clustering and asymmetry (BenSaïda 2021). After fitting the univariate GJR model to each return, the uniform filtered residuals are constructed using the Student’s *t* cumulative distribution function. The maximum likelihoods of the estimated marginals are presented in Table 4.^4^

**Table 4:** Estimation outputs of the marginals

G7 basket		BRICS basket	
BTC	6841.2	BTC	6841.2
CAD	14482.2	BRL	11751.1
EUR	14461.0	RUB	12108.2
GBP	13998.9	INR	15371.7
JPY	14354.5	CNH	16999.1
		ZAR	11837.9
Sum	64137.8	Sum	74909.2

This table reports the maximum likelihoods for the marginal estimations of the GJR model in Eq. (5)

### Vine copula construction

Given the uniformly filtered residuals from the previous step, the dependence is modeled using R-vine decomposition. This study uses the selection technique in Dißmann et al. (2013), where the nodes are connected with the largest dependence as measured by Kendall’s $\tau$, without any restriction on the tree structure nor on the bivariate copula family. The copula families included in this selection technique include the Independent (${\mathcal {I}}$), Gaussian (${\mathcal {N}}$), Student’s *t* (*t*), Clayton (${\mathcal {C}}$), Gumbel (${\mathcal {G}}$), Frank (${\mathcal {F}}$), Joe (${\mathcal {J}}$), Clayton-Gumbel (BB1), Joe-Hu (BB6), Joe-Clayton (BB7), Joe-Frank (BB8), Tawn type 1 (${\mathcal {T}}_1$) and Tawn type 2 (${\mathcal {T}}_2$) copulas, as well as their rotations by 90, 180, and 270 degrees (see, BenSaïda and Litimi 2021, foradetailedreview).

The copula estimation is performed in two steps. First, a sequential method is employed to jointly determine the tree structure and pair copulas in each node. Thereafter, the maximum likelihood estimation (MLE) using the tree specifications of the first step is used. This study selects the best copula that fits the data using AIC and BIC.

From the results provided in Table 5, for both the G7 and BRICS baskets, the R-vine structure is more appropriate than the C- or D-vine. The full estimation results are presented in Tables 9 and 10 in Appendix A for the G7 and BRICS markets, respectively.

**Table 5:** R-vine copula results for the full sample

	G7 basket		BRICS basket	
	Criteria	Tree structure	Criteria	Tree structure
No. of coefficients	18	R-vine	28	R-vine
Log-likelihood	1578.91		1382.93	
AIC	− 3121.82		− 2709.86	
BIC	− 3011.35		− 2538.02	

This table reports the selection criteria of the best vine decomposition for two sets of exchange rate returns. The G7 basket includes the Bitcoin and the G7 currency returns, and the BRICS basket includes the Bitcoin and the BRICS currency returns. For both G7 and BRICS baskets, the R-vine copula is the best choice. The reported log-likelihoods refer to the dependence structures excluding the marginals

**Fig. 4:**
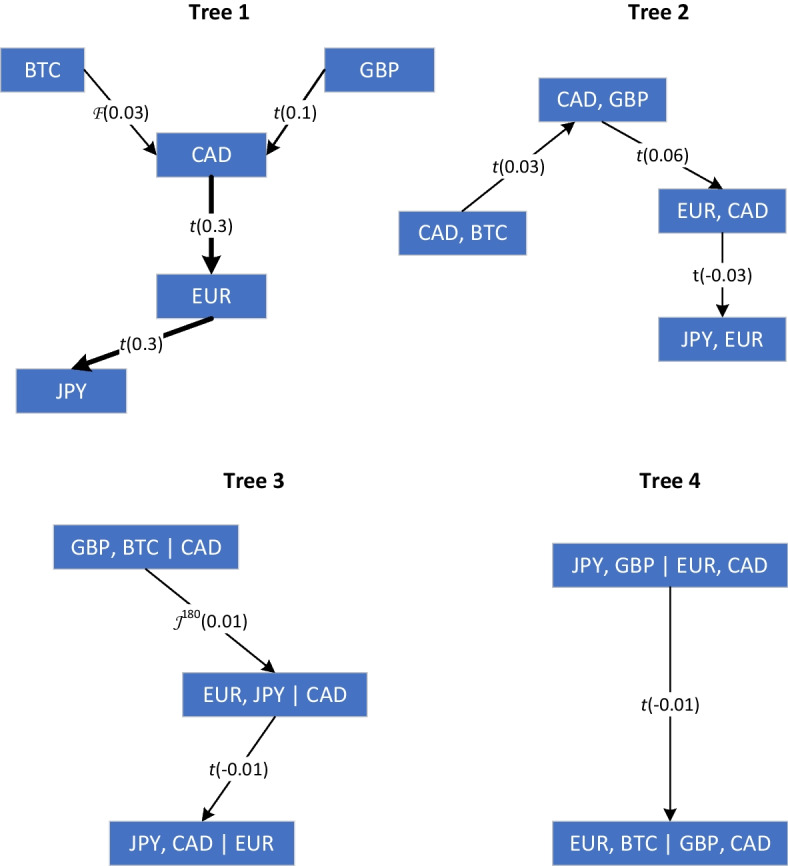
Estimated R-vine decomposition for G7 basket. The direction of the dependence transmission path is characterized by an arrow that indicates the pair copula and Kendall’s $\tau$ in parentheses. The strength of the dependence is illustrated with the thickness of the arrow

**Fig. 5:**
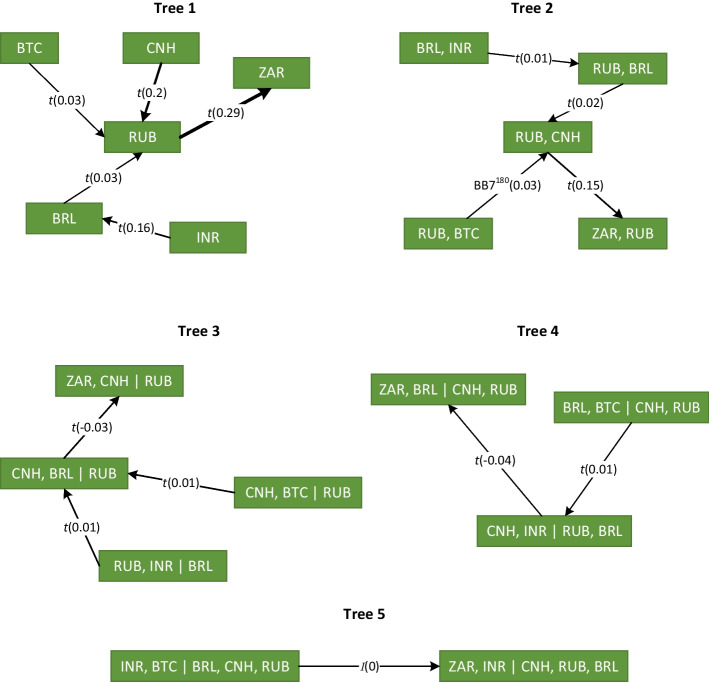
Estimated R-vine decomposition for BRICS basket. The direction of the dependence transmission path is characterized by an arrow that indicates the pair copula and Kendall’s $\tau$ in parentheses. The strength of the dependence is illustrated by the thickness of the arrow

Figures 4 and 5 exhibit the tree structures of the estimated vine copulas for G7 and BRICS baskets, respectively. These trees provide a clear view of the linkage between Bitcoin and the different exchange rates in each group of countries. The direction of the shock transmission path is indicated by the arrows. Moreover, the strength of the dependence between pairwise exchange rate returns, as measured by Kendall’s $\tau$, is illustrated with the thickness of the arrow that links the variables.

For developed G7 countries, the transmission path of the linkage between conventional currencies starts from the U.K. to Canada, then Europe, and ends up in Japan. The digital currency affects the Canadian dollar with weak dependency. Indeed, as argued by Chemkha et al. (2021a), cryptocurrency and conventional markets exhibit low integration in general, which encourages diversification.

For emerging BRICS countries, almost all currencies affect the Russian ruble, which in turn affects the South African rand. Moreover, in the case of G7 countries, the effect of the digital currency is relatively weak, suggesting that the Bitcoin market and BRICS exchange market are weakly integrated.

The findings suggest that the Bitcoin market is relatively isolated from the foreign exchange market during the entire sample period. Consequently, investors can reap the advantage of diversification by including the virtual currency in their international portfolios, in alignment with Bouri et al. (2017b), Chemkha et al. (2021a). On their side, policy makers may consider the specific characteristics of the cryptocurrency market to build more efficient decisions.

For comparison purposes, this study estimates the multivariate Student’s $t_\nu$ copula and the DCC model as benchmarks to model the dependence structure between BTC and G7 exchange rate returns, and BTC and BRICS currency rate returns. Table 6 summarizes the findings of the dependence structure for each basket. The degrees-of-freedom (*dof*) of the multivariate $t_\nu$ copula reflects the strength of the tail dependence. Low (high) *dof* indicates that the dependence between the variables is strong (weak) (BenMim and BenSaïda 2019).

**Table 6:** Estimation results of the benchmark models for the entire sample

	Multivariate $t_\nu$ copula		DCC model	
	G7 basket	BRICS basket	G7 basket	BRICS basket
*dof*	4.83	6.42	–	–
Log-likelihood	1161.6	1069.0	598.9	39.0
AIC	− 2301.2	− 2105.3	− 1191.7	− 72.0
BIC	− 2233.7	− 2007.1	− 1173.3	− 54.0

This table reports the selection criteria of the multivariate $t_\nu$ copula and the DCC model as benchmarks. The G7 basket includes Bitcoin and G7 currency returns, and the BRICS basket includes Bitcoin and BRICS currency returns. The reported log-likelihoods refer to the dependence structures excluding the marginals

According to AIC and BIC, the R-vine decomposition outperforms both the multivariate $t_\nu$ copula and the DCC model for both groups of countries. In contrast to vine copulas, the multivariate $t_\nu$ copula estimates the dependence structure as a whole without knowledge of the transmission path. Hence, the dependence between Bitcoin and other exchange rates is best modeled by providing sufficient information on the shock transmission path. Furthermore, the degrees-of-freedom clearly indicate that the developed G7 forex markets are more connected than the emerging BRICS forex markets.

### The effect of Bitcoin crashes

This section examines the effects of some documented Bitcoin crashes on the dependence structure on conventional exchange rates. Primarily, five crashes are investigated: (1) the Bitcoin meltdown of 2013, where the price dropped by 71% overnight and continued to drop until the end of that year. (2) The 2018 selloff, where the Bitcoin price fell by almost 65% in one month from January to February after an unprecedented boom in 2017, and continued to drop until the end of November. (3) The COVID-19 pandemic, which did not spare the cryptocurrency market. (4) The 2021 crash, where the Bitcoin price plunged from nearly $63,000 to less than $30,000 in just 100 days. (5) The 2022 crash and the Russia-Ukraine conflict that erupted with the invasion of Ukraine on February 24, 2022, which is still fueling extreme fear in the market.

**Fig. 6:**
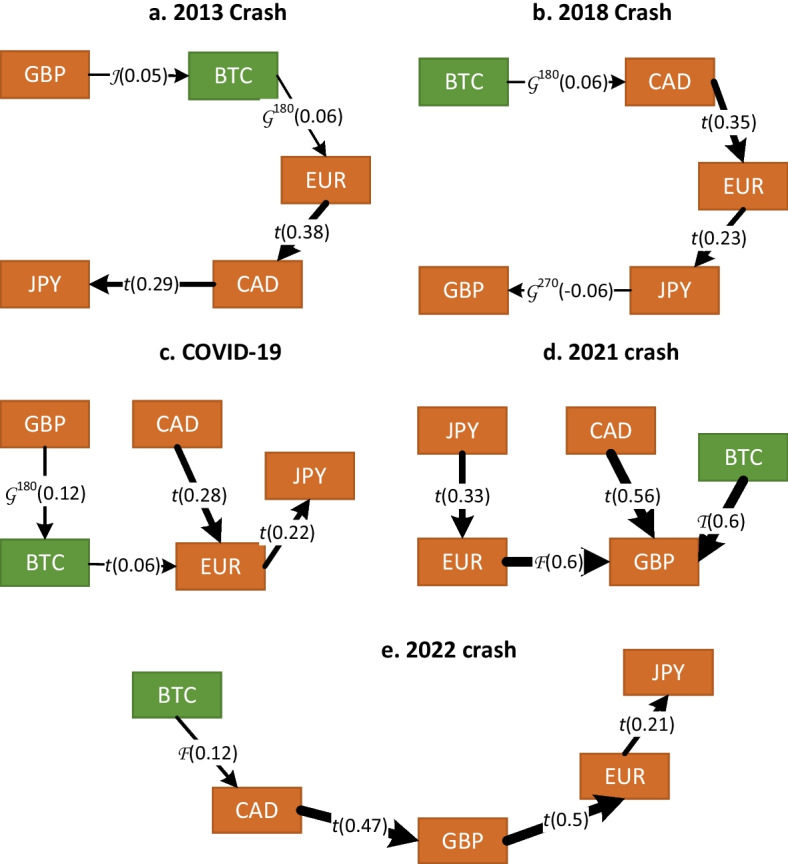
Estimated R-vine copulas (first trees) for G7 basket during five Bitcoin crashes. The direction of the dependence transmission path is characterized by an arrow that indicates the pair copula and Kendall’s $\tau$ in parentheses. The strength of the dependence is illustrated with the thickness of the arrow

**Fig. 7:**
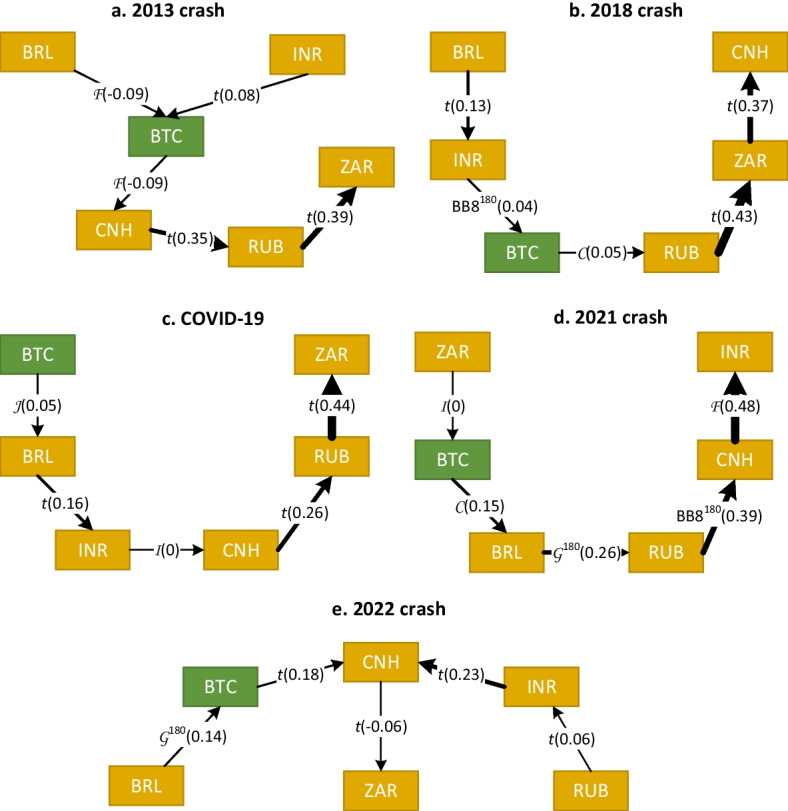
Estimated R-vine copulas (first trees) for BRICS basket during five Bitcoin crashes. The direction of the dependence transmission path is characterized by an arrow that indicates the pair copula and Kendall’s $\tau$ in parentheses. The strength of the dependence is illustrated with the thickness of the arrow

Table 7 reports the estimation results of the R-vine decomposition during the five crashes. Figures 6 and 7 illustrate the first trees for G7 basket and BRICS basket, respectively.

**Table 7:** R-vine copula results during Bitcoin crashes

	G7 basket					BRICS basket				
	2013 crash	2018 crash	COVID-19	2021 crash	2022 crash	2013 crash	2018 crash	COVID-19	2021 crash	2022 crash
Structure	D-vine	D-vine	R-vine	R-vine	D-vine	R-vine	D-vine	D-vine	D-vine	R-vine
Sample size	244	334	294	142	198	244	334	294	142	198
No. of coefficients	15	13	15	13	17	20	19	18	14	20
Log-likelihood	157.09	166.88	133.82	210.28	250.91	121.47	234.13	213.65	98.24	89.68
AIC	− 284.18	− 307.75	− 237.64	− 394.57	− 467.82	− 202.95	− 430.26	− 391.31	− 168.48	− 139.35
BIC	− 231.73	− 258.21	− 182.38	− 356.14	− 411.83	− 133.00	− 357.85	− 325.00	− 127.1	− 73.49

This table reports the selection criteria of the best vine decomposition for two sets of exchange rate returns during the Bitcoin crashes. The G7 basket includes Bitcoin and G7 currency returns, and the BRICS basket includes Bitcoin and BRICS currency returns. The reported log-likelihoods refer to the dependence structures excluding the marginals

**Table 8:** Estimation results of the benchmark models during Bitcoin crashes

	G7 basket					BRICS basket				
	2013 crash	2018 crash	COVID-19	2021 crash	2022 crash	2013 crash	2018 crash	COVID-19	2021 crash	2022 crash
	*Panel A: multivariate* $t_\nu$ **copula**									
*dof*	4.91	4.92	4.52	4.15	3.67	7.38	6.09	5.76	4.96	5.01
Log-likelihood	112.20	119.80	108.50	180.20	220.80	80.33	179.60	175.70	88.85	82.71
AIC	− 202.46	− 217.70	− 195.03	− 338.47	− 419.67	− 128.66	− 363.16	− 319.38	− 145.71	− 133.41
BIC	− 163.99	− 175.77	− 154.51	− 305.96	− 383.44	− 72.70	− 302.18	− 260.44	− 98.41	− 80.72
	*Panel B: DCC model*									
Log-likelihood	73.98	88.62	− 22.05	146.76	41.42	− 132.91	− 9.04	− 62.07	25.71	17.20
AIC	− 141.96	− 171.24	50.10	− 287.51	− 76.84	271.82	24.09	130.14	− 45.41	− 28.40
BIC	− 131.47	− 159.80	61.15	− 278.65	− 66.98	282.31	35.52	141.19	− 36.54	− 18.53

This table reports the selection criteria of the multivariate $t_\nu$ copula (Panel A) and DCC model (Panel B) as benchmarks during the Bitcoin crashes. The G7 basket includes Bitcoin and G7 currency returns, and the BRICS basket includes Bitcoin and BRICS currency returns. The reported log-likelihoods refer to the dependence structures excluding the marginals

This study compares the R-vine estimation results during Bitcoin crashes with the multivariate $t_\nu$ copula (Panel A) and DCC model (Panel B) reported in Table 8. According to the selection criteria, this study confirms the previous findings that the R-vine decomposition better describes the linkage between the virtual currency and the fiat currencies of the developed G7 countries and emerging BRICS countries.

#### Impact of the 2013 Bitcoin crash

The Bitcoin meltdown in 2013 was a landmark year in the history of Bitcoin, where it experienced the first major speculative turmoil (Cheah and Fry 2015). In April 2013, the price of Bitcoin dropped from $233 to around $67 overnight, a massive decline of 71% in just 12 h. This drastic slump was ascribed to the fact that Bitcoin rubbed shoulders with mainstream currencies for the first time. In addition, in April, the funds of the German exchange platform Bitcoin-24 were blocked by authorities, resulting in its effective disappearance. In May, Mt. Gox also saw a portion of its funds (over $5 million) blocked by U.S. authorities due to a financial dispute, without filing for bankruptcy.

After April 2013, Bitcoin price fluctuated around $120 and suddenly soared to a high of $1,150 in late November, tumbling back to half that in mid-December. Therefore, this study specifies the 2013 Bitcoin crash as the period from May 2013 to December 2013.

Figures 6a and 7a indicate that the effect of Bitcoin on the G7 and BRICS baskets has increased during the 2013 crash. Kendall’s $\tau$ moved from 0.03 during the entire period to 0.06 and $-0.09$ for G7 and BRICS, respectively. However, the relative increase in connectedness remains moderate. Furthermore, the types of R-vine tree structures have changed to a D-vine for the G7 basket and to an R-vine for the BRICS basket. Variable ordering has also been modified and BTC is no longer at the origin of the trees.

This may be explained by the fact that Bitcoin exhibits a low correlation with traditional assets like currencies in normal periods as well as in times of financial crises, as suggested by Baur and Lucey (2015). Moreover, Dyhrberg (2016), Bouri et al. (2017a), Bouri et al. (2017b) outline the capability of Bitcoin to be a hedge and safe haven against several financial assets, including major world equities, gold, bonds, oil, currencies, and commodities. Consequently, Bitcoin is considered an effective diversifier during both normal and crisis periods and may then be used to considerably reduce the risk of investment portfolios, especially for risk-averse investors.

#### Impact of the 2018 Bitcoin crash

After reaching its highest ever recorded price on December 16, 2017, the Bitcoin price fell by approximately 65% in one month from January to February 2018. The cryptocurrency selloff started in January 2018 and continued until November 2018. By the end of November 2018, the Bitcoin price had fallen by over 80% of its peak, which means as low as $5500 since the previous year’s boom.

For the G7 basket, Fig. 6b shows that the best tree structure is a D-vine copula with BTC at the origin; however, the effect of the digital currency remains weak relative to conventional currencies. Kendall’s $\tau$ moved from 0.03 during the entire period to 0.06 and 0.05 for G7 and BRICS, respectively, which indicates a slight increase in the strength of the dependence between Bitcoin and other currencies during the 2018 selloff. Similarly, for the BRICS basket, Fig. 7b reveals that the best copula decomposition is a D-vine; however, BTC is not at the origin of the tree, and its dependence on other currencies remains weak.

Consequently, the dependence between Bitcoin and fiat currencies in the G7 and BRICS markets increased slightly in intensity during the 2018 crash. These findings extend the results of Ji et al. (2018), where Bitcoin is relatively isolated from other financial assets during stable periods and market integration between the digital currency and other assets varies over time.

#### Impact of the COVID-19 pandemic

COVID-19 erupted in China in late December 2019, and was declared a global pandemic in March 2020 and termed the Great Lockdown by the IMF (Le et al. 2021). Its severity has drastically impacted financial markets around the globe, where investors have engaged in panic-sold trading out of fear (Akhtaruzzaman et al. 2021). The decrease in the value of Bitcoin continued until October 2020, when it was worth approximately $13,200. Thereafter, it started to regain its value to surpass its previous all-time high in November 2020. According to Table 8, the *dof* for the G7 and BRICS baskets was lower during COVID-19 than the previous Bitcoin crashes. Therefore, the linkage between BTC and other groups of currencies has increased during the pandemic.

The inspection of the R-vine trees in Figs. 6c and 7c reveals that the connectedness between Bitcoin and traditional currencies increased slightly, with Kendall’s $\tau$ moving from 0.03 during the full sample to 0.06 and 0.05 for G7 and BRICS, respectively. Nevertheless, the effect of the digital currency on other conventional currencies remains weak.

Research on the classification of Bitcoin as a safe haven during the COVID-19 pandemic is controversial. For instance, Conlon and McGee (2020) employed a four-moment Value-at-Risk method, and Corbet et al. (2020) calculated linear correlations of the data to cast doubt on the ability of the digital currency to provide shelter during the COVID-19 pandemic and concluded that Bitcoin is losing its position as a safe haven. However, both methods have been highly criticized in the literature due to their poor performance in detecting complex dependence dynamics across markets (Chemkha et al. 2021a). On the other hand, Bouri et al. (2020), Goodell and Goutte (2021) used wavelet analysis and Dwita Mariana et al. (2020) employed a multivariate GARCH model, concluding that Bitcoin exhibits safe haven properties during the COVID-19 pandemic.

#### Impact of the 2021 Bitcoin crash

In early 2021, Bitcoin witnessed a spectacular boom in which the price soared to more than $63,000 in April. This drastic increase can be attributed mainly to two reasons. First, on February 8, 2021, Tesla announced that it had bought $1.5 billion worth of Bitcoin, and it started accepting the digital currency as a payment method for its products. Second, on April 14, 2021, the cryptocurrency exchange platform Coinbase went public on NASDAQ (Chemkha et al. 2021b). Nevertheless, on May 12, 2021, Tesla stopped accepting Bitcoin as a payment method, and China’s central bank reiterated that cryptocurrencies cannot be accepted as a payment method. As a result, Bitcoin price plummeted from nearly $58,000 to below $38,000 in just 10 days. Indeed, amid the market-wide price crash, almost all cryptocurrencies experienced a double-digit percentage decrease in their prices. The recovery surged around September 2021 when El Salvador declared Bitcoin as a legal tender, with many investors wondering which country would be next. Bitcoin reached a new unbroken all-time high of $68,000 on November 8, 2021.

Figures 6d and 7d reveal that the intensity of the effect of Bitcoin on other currencies has substantially increased to reach a Kendall’s $\tau$ of 0.6 and 0.15 for G7 and BRICS, respectively. The strongest effect is observed for the British pound, where the cryptocurrency market became highly integrated with the conventional foreign exchange market for the first time. The traditional role of the digital currency as a safe haven or hedge during the 2021 crash should be reconsidered.

#### Impact of the 2022 crash and the Russia-Ukraine conflict

From late 2021 to early 2022, Bitcoin slipped into a bear market and continued to plunge below $20,000 in September 2022. The digital market crash in 2022 was a storm of several unfortunate events. Mainly, the invasion of Ukraine on February 24, 2022 that did not spare the cryptocurrency. Indeed, the ongoing Russia-Ukraine conflict is still fueling extreme fear in the market, and investors are seeking liquidity, which explains massive selloffs by major holders, as argued by Khalfaoui et al. (2022). Moreover, the Terra-Luna crypto asset crash was due to a crisis of algorithmic stablecoins.^5^

During this crisis, Table 8 report that the *dof* for the G7 basket is the lowest among other crisis periods, suggesting a strong connectedness among the G7 currencies, even stronger than during the COVID-19 pandemic. Figure 6e reveals that the optimal tree structure is a D-vine with Bitcoin at its origin, but with a reduced linkage compared to the 2021 crash. Moreover, the optimal tree structure for the BRICS basket in Fig. 7e is an interesting R-vine, where the Russian ruble affects the Chinese yuan through the Indian rupee, which in turn is affected by Bitcoin and the Brazilian real. As argued by Umar et al. (2022), the connectedness among Bitcoin and G7 and BRICS baskets is affected by the war.

## Conclusion

This study investigates the connectedness between Bitcoin and the forex currencies of two groups of countries: developed G7 and emerging BRICS. The study includes five major documented crashes of the cryptocurrency. The regular (R) vine decomposition is employed to further provide sufficient information on the shock transmission path.

The results highlight the weak dependency between Bitcoin and the G7 currencies, as well as the BRICS exchange rates over the entire period under study. This is consistent with the previous literature that finds the cryptocurrency market is relatively isolated and can provide shelter to international investors from turmoil in traditional markets. However, the connectedness with the digital currency increased during the 2021 and 2022 crashes. Furthermore, for both the G7 and BRICS baskets, the vine copulas outperform the multivariate $t_\nu$ copula and the dynamic conditional correlation (DCC) model, set as benchmarks. Hence, the dependence between Bitcoin and other exchange rates is best modeled by providing sufficient information on the shock transmission path.

Alternatively, this study analyzes the changes in the dependence structure during five major Bitcoin crashes: the meltdown of 2013, selloff of 2018, COVID-19 pandemic, 2021 crash, and Russia-Ukraine conflict in 2022. The dependence structure changed during each crash, and the effect of Bitcoin on other exchange rates increased slightly, except during the 2021 and 2022 crashes, when the digital currency started to have a considerable effect on the conventional forex markets. The cross-market linkages during the last two crashes of 2021 and 2022 are stronger for developed G7 countries than for emerging BRICS countries. Indeed, starting from 2021, the price of Bitcoin has reached impressive records and attracted the attention of international investors around the globe. This increase in the linkage between the cryptocurrency and fiat currencies during the last periods of turmoil may be the end of the market isolation of Bitcoin.

Future studies could extend the sample to include other cryptocurrencies and conventional currencies to examine the global connectedness across the digital and foreign exchange markets. Researchers can investigate the efficiency of a mixed portfolio that includes digital and conventional currencies for optimum risk management.

## Data Availability

The datasets used and/or analyzed during the current study are available from the corresponding author on reasonable request.

## Appendix A: R-vine full estimation results

See Tables 9 and 10.

**Table 9:** Full estimation results of the R-vine copula for G7 basket

Tree	Edge	Copula	Copula parameters		Kendall’s $\tau$	Tail dependence	
			$\theta$ ($\rho$ for *t* or ${\mathcal {N}}$)	$\delta$ ($\nu$ for *t*)		$\lambda _U$	$\lambda _L$
$T_1$	2,1	${\mathcal {F}}$	0.29 (0.10)***	–	0.03	0	0
	2,4	*t*	0.15 (0.02)***	3.72 (0.30)***	0.10	0.12	0.12
	3,2	*t*	0.45 (0.02)***	2.09 (0.10)***	0.30	0.36	0.36
	5,3	*t*	0.46 (0.02)***	2.03 (0.09)***	0.30	0.37	0.37
$T_2$	4,1 | 2	*t*	0.04 (0.02)**	20.9 (7.11)***	0.03	0.00	0.00
	3,4 | 2	*t*	0.09 (0.02)***	7.36 (1.03)***	0.06	0.03	0.03
	5,2 | 3	*t*	− 0.04 (0.02)**	2.68 (0.15)***	− 0.03	0.12	0.12
$T_3$	3,1 | 4,2	${\mathcal {J}}^{180}$	1.02 (0.01)***	–	0.01	0	0.03
	5,4 | 3,2	*t*	− 0.01 (0.02)	13.4 (2.91)***	− 0.01	0.00	0.00
$T_4$	5,1 | 3,4,2	*t*	− 0.01 (0.02)	27.8 (10.1)***	− 0.01	0	0

This table reports the full estimation results of the R-vine decomposition for Bitcoin and the developed G7 exchange rates. The best structure is a regular (R) vine. Standard errors of the estimated parameters are in parentheses. The edges are 1 $\leftrightarrow$ BTC, 2 $\leftrightarrow$ CAD, 3 $\leftrightarrow$ EUR, 4 $\leftrightarrow$ GBP, and 5 $\leftrightarrow$ JPY. The employed copulas are Independent (${\mathcal {I}}$), Gaussian (${\mathcal {N}}$), *t*, Clayton (${\mathcal {C}}$), Gumbel (${\mathcal {G}}$), Frank (${\mathcal {F}}$), Joe (${\mathcal {J}}$), BB1, BB6, BB7, BB8, Tawn 1 (${\mathcal {T}}_1$), and Tawn 2 (${\mathcal {T}}_2$). Rotation degrees are indicated as superscripts. The log-likelihood of this structure is 1578.91, the AIC: $-$3121.82, and the BIC: $-$3011.35

*** Statistically significant at the 1% level

** Statistically significant at the 5% level

**Table 10:** Full estimation results of the R-vine copula for BRICS basket

Tree	Edge	Family	Copula parameters		Kendall’s $\tau$	Tail dependence	
			$\theta$ ($\rho$ for *t* or ${\mathcal {N}}$)	$\delta$ ($\nu$ for *t*)		$\lambda _U$	$\lambda _L$
$T_1$	3,1	*t*	0.05 (0.02)**	18.0 (5.72)***	0.03	0.00	0.00
	2,4	*t*	0.25 (0.02)***	2.69 (0.16)***	0.16	0.22	0.22
	3,2	*t*	0.05 (0.02)**	6.33 (0.84)***	0.03	0.04	0.04
	3,5	*t*	0.31 (0.02)***	3.15 (0.19)***	0.20	0.21	0.21
	6,3	*t*	0.44 (0.02)***	2.35 (0.12)***	0.29	0.33	0.33
$T_2$	5,1 | 3	BB7^180^	1.01 (0.01)***	0.05 (0.02)**	0.03	0.00	0.02
	3,4 | 2	*t*	0.02 (0.02)	9.55 (1.59)***	0.02	0.01	0.01
	5,2 | 3	*t*	0.03 (0.02)	8.43 (1.26)***	0.02	0.01	0.01
	6,5 | 3	*t*	0.24 (0.02)***	3.31 (0.22)***	0.15	0.17	0.17
$T_3$	2,1 | 5,3	*t*	0.02 (0.02)	17.8 (5.60)***	0.01	0.00	0.00
	5,4 | 3,2	*t*	0.02 (0.02)	24.4 (8.87)***	0.01	0.00	0.00
	6,2 | 5,3	*t*	− 0.04 (0.02)**	19.9 (6.65)***	− 0.03	0.00	0.00
$T_4$	4,1 | 2,5,3	*t*	0.02 (0.02)	23.5 (8.32)***	0.01	0.00	0.00
	6,4 | 5,3,2	*t*	-0.06 (0.02)***	19.7 (5.88)***	− 0.04	0.00	0.00
$T_5$	6,1 | 4,2,5,3	${\mathcal {I}}$	–	–	0.00	0	0

This table reports the full estimation results of the R-vine decomposition for Bitcoin and the emerging BRICS exchange rates. The best structure is a regular (R) vine. Standard errors of the estimated parameters are in parentheses. The edges are 1 $\leftrightarrow$ BTC, 2 $\leftrightarrow$ BRL, 3 $\leftrightarrow$ RUB, 4 $\leftrightarrow$ INR, 5 $\leftrightarrow$ CNH, and 6 $\leftrightarrow$ ZAR. The employed copulas are Independent (${\mathcal {I}}$), Gaussian (${\mathcal {N}}$), *t*, Clayton (${\mathcal {C}}$), Gumbel (${\mathcal {G}}$), Frank (${\mathcal {F}}$), Joe (${\mathcal {J}}$), BB1, BB6, BB7, BB8, Tawn 1 (${\mathcal {T}}_1$), and Tawn 2 (${\mathcal {T}}_2$). Rotation degrees are indicated as superscripts. The log-likelihood of this structure is 1382.93, the AIC: $-$2709.86, and the BIC: $-$2538.02

*** Statistically significant at the 1% level

** Statistically significant at the 5% level

## Abbreviations

forex: Foreign exchange
R-vine: Regular vine
C-vine: Canonical vine
D-vine: Drawable vine
DCC: Dynamic conditional correlation
*dof*: Degrees-of-freedom
*cdf*: Cumulative distribution function
*pdf*: Probability distribution function
AIC: Akaike information criterion
BIC: Bayesian information criterion

## Footnotes

1 In this representation, the parameters correspond to the elements in the symmetric correlation matrix ${\mathbf{R}}$ and the degrees-of-freedom $\nu$; hence, the number of coefficients to estimate is ${d \left( d-1 \right) }/{2} + 1$.

2 For the DCC(1,1) model under the multivariate Student’s *t* distribution, the dependence structure contains three parameters to estimate: *a*, *b*, and the degrees-of-freedom $\nu$.

3 The comparison is conducted between the R-vine model and the benchmark models for the same basket and for the same sample period.

4 All empirical estimations of the marginals, R-vine copula, multivariate $t_\nu$ copula, DCC model are conducted using the programming language R and the packages **rmgarch** for multivariate GARCH models, **rugarch** for univariate GARCH models, **VineCopula** for R-vines, **copula** for the multivariate $t_\nu$ copula, and freely available from r-project.org.

5 The algorithmic stablecoin of Terra-Luna is designed to support a stable 1:1 peg with the U.S. dollar through blockchain algorithms rather than equivalent cash reserves.

## References

[CR1] Aharon DY, Umar Z, Vo XV (2021). Dynamic spillovers between the term structure of interest rates, bitcoin, and safe-haven currencies. Financ Innov.

[CR2] Akhtaruzzaman M, Boubaker S, Sensoy A (2021). Financial contagion during COVID-19 crisis. Financ Res Lett.

[CR3] Baur DG, Dimpfl T (2021). The volatility of Bitcoin and its role as a medium of exchange and a store of value. Empir Econ.

[CR4] Baur DG, Lucey BM (2015). Is gold a hedge or a safe haven? An analysis of stocks, bonds and gold. Financ Rev.

[CR5] BenMim I, BenSaïda A (2019). Financial contagion across major stock markets: a study during crisis episodes. N Am J Econ Financ.

[CR6] BenSaïda A (2018). The contagion effect in European sovereign debt markets: a regime-switching vine copula approach. Int Rev Financ Anal.

[CR7] BenSaïda A (2021). The good and bad volatility: a new class of asymmetric heteroskedastic models. Oxford Bull Econ Stat.

[CR8] BenSaïda A, Litimi H (2021). Financial contagion across G10 stock markets: a study during major crises. Int J Financ Econ.

[CR9] Blau BM (2017). Price dynamics and speculative trading in bitcoin. Res Int Bus Financ.

[CR10] Böhme R, Christin N, Edelman B, Moore T (2015). Bitcoin: economics, technology, and governance. J Econ Perspect.

[CR11] Bouoiyour J, Selmi R, Tiwari AK (2015). Is Bitcoin business income or speculative foolery? New ideas through an improved frequency domain analysis. Ann Financ Econ.

[CR12] Bouri E, Jalkh N, Molnár P, Roubaud D (2017). Bitcoin for energy commodities before and after the December 2013 crash: diversifier, hedge or safe haven?. Appl Econ.

[CR13] Bouri E, Molnár P, Azzi G, Roubaud D, Hagfors LI (2017). On the hedge and safe haven properties of Bitcoin: Is it really more than a diversifier?. Financ Res Lett.

[CR14] Bouri E, Shahzad SJH, Roubaud D, Kristoufek L, Lucey B (2020). Bitcoin, gold, and commodities as safe havens for stocks: New insight through wavelet analysis. Q Rev Econ Financ.

[CR15] Brandvold M, Molnár P, Vagstad K, Andreas Valstad OC (2015). Price discovery on Bitcoin exchanges. J Int Finan Markets Inst Money.

[CR16] Brière M, Oosterlinck K, Szafarz A (2015). Virtual currency, tangible return: portfolio diversification with bitcoin. J Asset Manag.

[CR17] Carrick J (2016). Bitcoin as a complement to emerging market currencies. Emerg Mark Financ Trade.

[CR18] Cheah ET, Fry J (2015). Speculative bubbles in Bitcoin markets? An empirical investigation into the fundamental value of Bitcoin. Econ Lett.

[CR19] Chemkha R, BenSaïda A, Ghorbel A (2021). Connectedness between cryptocurrencies and foreign exchange markets: implication for risk management. J Multinatl Financ Manag.

[CR20] Chemkha R, BenSaïda A, Ghorbel A, Tayachi T (2021). Hedge and safe haven properties during COVID-19: evidence from Bitcoin and gold. Q Rev Econ Financ.

[CR21] Ciaian P, Rajcaniova M, Kancs D (2016). The economics of Bitcoin price formation. Appl Econ.

[CR22] Conlon T, McGee R (2020). Safe haven or risky hazard? Bitcoin during the Covid-19 bear market. Financ Res Lett.

[CR23] Corbet S, McHugh G, Meegan A (2017). The influence of central bank monetary policy announcements on cryptocurrency return volatility. Invest Manag Financ Innov.

[CR24] Corbet S, Meegan A, Larkin C, Lucey B, Yarovaya L (2018). Exploring the dynamic relationships between cryptocurrencies and other financial assets. Econ Lett.

[CR25] Corbet S, Larkin C, Lucey B (2020). The contagion effects of the COVID-19 pandemic: evidence from gold and cryptocurrencies. Financ Res Lett.

[CR26] Dahir AM, Mahat F, Amin Noordin BA, Hisyam Ab Razak N (2020). Dynamic connectedness between Bitcoin and equity market information across BRICS countries: evidence from TVP-VAR connectedness approach. Int J Manag Financ.

[CR27] Dißmann J, Brechmann E, Czado C, Kurowicka D (2013). Selecting and estimating regular vine copulae and application to financial returns. Comput Stat Data Anal.

[CR28] Dwita Mariana C, Ekaputra IA, Husodo ZA (2020). Are Bitcoin and Ethereum safe-havens for stocks during the COVID-19 pandemic?. Financ Res Lett.

[CR29] Dyhrberg AH (2016). Hedging capabilities of bitcoin: is it the virtual gold?. Financ Res Lett.

[CR30] Fang F, Ventre C, Basios M, Kanthan L, Martinez-Rego D, Wu F, Li L (2022). Cryptocurrency trading: a comprehensive survey. Financ Innov.

[CR31] Garcia D, Tessone CJ, Mavrodiev P, Perony N (2014). The digital traces of bubbles: feedback cycles between socio-economic signals in the Bitcoin economy. J R Soc Interface.

[CR32] Glosten LR, Jagannathan R, Runkle DE (1993). On the relation between the expected value and the volatility of the nominal excess return on stocks. J Financ.

[CR33] Goodell JW, Goutte S (2021). Co-movement of COVID-19 and Bitcoin: evidence from wavelet coherence analysis. Financ Res Lett.

[CR34] Goodell JW, Alon I, Chiaramonte L, Dreassi A, Paltrinieri A, Piserà S (2022). Risk substitution in cryptocurrencies: evidence from BRICS announcements. Emerg Markets Rev.

[CR35] Hanley BP (2018) The false premises and promises of bitcoin. arXiv:1312.2048

[CR36] Huang GY, Gau YF, Wu ZX (2022). Price discovery in fiat currency and cryptocurrency markets. Financ Res Lett.

[CR37] Ji Q, Bouri E, Gupta R, Roubaud D (2018). Network causality structures among Bitcoin and other financial assets: a directed acyclic graph approach. Q Rev Econ Financ.

[CR38] Kang KY, Lee S (2022). Money, Bitcoin, and monetary policy. J Money Credit Banking.

[CR39] Khalfaoui R, Gozgor G, Goodell JW (2022). Impact of Russia-Ukraine war attention on cryptocurrency: evidence from quantile dependence analysis. Financ Res Lett.

[CR40] Kristoufek L (2015). What are the main drivers of the bitcoin price? Evidence from wavelet coherence analysis. PLoS ONE.

[CR41] Kurka J (2019). Do cryptocurrencies and traditional asset classes influence each other?. Financ Res Lett.

[CR42] Le TH, Do HX, Nguyen DK, Sensoy A (2021). Covid-19 pandemic and tail-dependency networks of financial assets. Financ Res Lett.

[CR43] Levulytè L, Šapkauskiené A (2021). Cryptocurrency in context of fiat money functions. Q Rev Econ Financ.

[CR44] Mensi W, Shafiullah M, Vo XV, Kang SH (2021). Volatility spillovers between strategic commodity futures and stock markets and portfolio implications: evidence from developed and emerging economies. Resour Policy.

[CR45] Nakamoto S (2008) Bitcoin: a peer-to-peer electronic cash system. http://bitcoin.org/bitcoin.pdf

[CR46] Ning C (2010). Dependence structure between the equity market and the foreign exchange market: a copula approach. J Int Money Financ.

[CR47] Palazzi RB, de Souza-Raimundo G, Klotzle MC (2021). The dynamic relationship between bitcoin and the foreign exchange market: a nonlinear approach to test causality between bitcoin and currencies. Financ Res Lett.

[CR48] Polasik M, Piotrowska AI, Wisniewski TP, Kotkowski R, Lightfoot G (2015). Price fluctuations and the use of Bitcoin: an empirical inquiry. Int J Electron Commer.

[CR49] Popper N (2016). Digital gold: the untold story of Bitcoin.

[CR50] Rognone L, Hyde S, Zhang SS (2020). News sentiment in the cryptocurrency market: an empirical comparison with forex. Int Rev Financ Anal.

[CR51] Rogojanu A, Badea L (2014). The issue of competing currencies: case study Bitcoin. Theor Appl Econ.

[CR52] Salisu AA, Cuñado J, Gupta R (2022). Geopolitical risks and historical exchange rate volatility of the BRICS. Int Rev Econ Financ.

[CR53] Sebastião H, Godinho P (2021). Forecasting and trading cryptocurrencies with machine learning under changing market conditions. Financ Innov.

[CR54] Shahzad SJH, Balli F, Naeem MA, Hasan M, Arif M (2022). Do conventional currencies hedge cryptocurrencies?. Q Rev Econ Financ.

[CR55] Shahzad SJH, Bouri E, Rehman MU, Roubaud D (2022). The hedge asset for BRICS stock markets: Bitcoin, gold or VIX. World Econ.

[CR56] Shubik M (2014) Simecs, ithaca hours, berkshares, bitcoins and walmarts. Tech. Rep. 1947, Cowles Foundation Discussion Paper, 10.2139/ssrn.2435902

[CR57] Sklar A (1959). Fonctions de répartition à n dimensions et leurs marges. Publications de l’Institut de Statistique de L’Université de Paris.

[CR58] Umar Z, Polat O, Choi S, Teplova T (2022). The impact of the Russia-Ukraine conflict on the connectedness of financial markets. Financ Res Lett.

[CR59] Urquhart A, Zhang H (2019). Is Bitcoin a hedge or safe haven for currencies? An intraday analysis. Int Rev Financ Anal.

[CR60] Virk N (2022). Bitcoin and integration patterns in the forex market. Financ Res Lett.

[CR61] Woo D, Gordon I, Iralov V (2013) Bitcoin: a first assessment. Tech. rep., Bank of America. Merrill Lynch, https://www.aargauerzeitung.ch/asset_document/i/127472557/download

[CR62] Xu M, Chen X, Kou G (2019). A systematic review of blockchain. Financ Innov.

[CR63] Xu Y, Lien D (2022). COVID-19 and currency dependences: empirical evidence from BRICS. Financ Res Lett.

[CR64] Zhang Y, Hamori S (2022). A connectedness analysis among BRICS’s geopolitical risks and the US macroeconomy. Econ Anal Policy.

